# Low energy irradiation of narrow-range UV-LED prevents osteosarcopenia associated with vitamin D deficiency in senescence-accelerated mouse prone 6

**DOI:** 10.1038/s41598-020-68641-8

**Published:** 2020-07-17

**Authors:** Kazuya Makida, Yoshihiro Nishida, Daigo Morita, Satoshi Ochiai, Yoshitoshi Higuchi, Taisuke Seki, Kunihiro Ikuta, Naoki Ishiguro

**Affiliations:** 1 Department of Orthopaedic Surgery, Nagoya University Graduate School of Medicine, 65 Tsurumai-cho, Showa-ku, Nagoya, 466-8550 Japan; 2 Department of Rehabilitation Medicine, Nagoya University Graduate School of Medicine, Nagoya, Japan

**Keywords:** Skin diseases, Malnutrition, Metabolic bone disease, Ageing, Bone, Calcium and vitamin D, Inorganic LEDs

## Abstract

Deficiency of vitamin D is an important cause of osteosarcopenia. The purpose of this study is to examine the effects of low energy narrow-range UV-LED on osteosarcopenia in animal models of senescence-accelerated mouse prone 6 (SAMP6). Preliminary experiments specified the minimum irradiance intensity and dose efficacy for vitamin D production (316 nm, 0.16 mW/cm^2^, 1,000 J/m^2^). we set a total of 4 groups (n = 8 per group); vitamin D-repletion without UV irradiation (Vit.D+UV−), vitamin D-repletion with UV irradiation (Vit.D+UV +), vitamin D-deficiency without UV irradiation, (Vit.D−UV−), and vitamin D-deficiency with UV irradiation (Vit.D−UV +). Serum levels of 25(OH)D at 28 and 36 weeks of age were increased in Vit.D−UV+ group as compared with Vit.D−UV− group. Trabecular bone mineral density on micro-CT was higher in Vit.D−UV+ group than in Vit.D−UV− group at 36 weeks of age. In the histological assay, fewer osteoclasts were observed in Vit.D−UV+ group than in Vit.D−UV− group. Grip strength and muscle mass were higher in Vit.D−UV+ group than in Vit.D−UV− group at 36 weeks of age. Signs of severe damage induced by UV irradiation was not found in skin histology. Low energy narrow-range UV irradiation may improve osteosarcopenia associated with vitamin D deficiency in SAMP6.

## Introduction

Osteosarcopenia is a novel concept denoting the co-existence of decreased density of both bone and muscle, namely osteoporosis and sarcopenia^1,2^. This condition promotes falls and insufficiency fractures that impede the activities of daily living (ADL) of elderly persons^3,4^. This impairment of ADL is becoming an increasingly serious social problem, associated particularly with the aging of society seen in developed countries. Vitamin D is one of the most important molecules associated with osteosarcopenia^5^. However, it has been reported that the elderly are consistently deficient in vitamin D^6–8^. Adequate supplementation of vitamin D for elderly persons with low cost and high safety is an urgent issue for all rapidly aging societies.

It has been reported that 90% of vitamin D is produced by the skin with UV irradiation from sunlight^9,10^. This makes it crucial to devise effective and safe UV irradiation for vitamin D supplementation. We previously reported that short-range UV irradiation using LED was effective for elevation of serum vitamin D levels and prevention of bone brittleness in an animal model of vitamin D deficiency^11,12^. In that study, irradiation with UV-LED was 0.54 mW/cm^2^, and the wavelength of LED was 305 nm, which was estimated to be equivalent to 6.75 points in the UV index specified by WHO, an amount of irradiation considered to constitute a high risk of harm to the human body.

Recently, several studies have clarified that vitamin D deficiency is associated with reduced muscle strength and volume, namely sarcopenia^13,14^. However, the relationship between muscle and vitamin D supply by UV irradiation has not been investigated.

We hypothesized that if the dose of UV irradiation by LED (UV-LED) could be reduced, but still increase bone mass and muscle mass, a treatment device characterized by low cost and high safety could be developed. The purpose of this study is to determine the minimal irradiance intensity and dose of short-range UV-LED that would be effective in supplying sufficient levels of serum vitamin D, and to examine the effects of UV-LED with a determined irradiance and dose on osteosarcopenia in animal models of senescence-accelerated mouse prone 6 (SAMP6).

## Results

### Preliminary experiments

**Figure 1:**
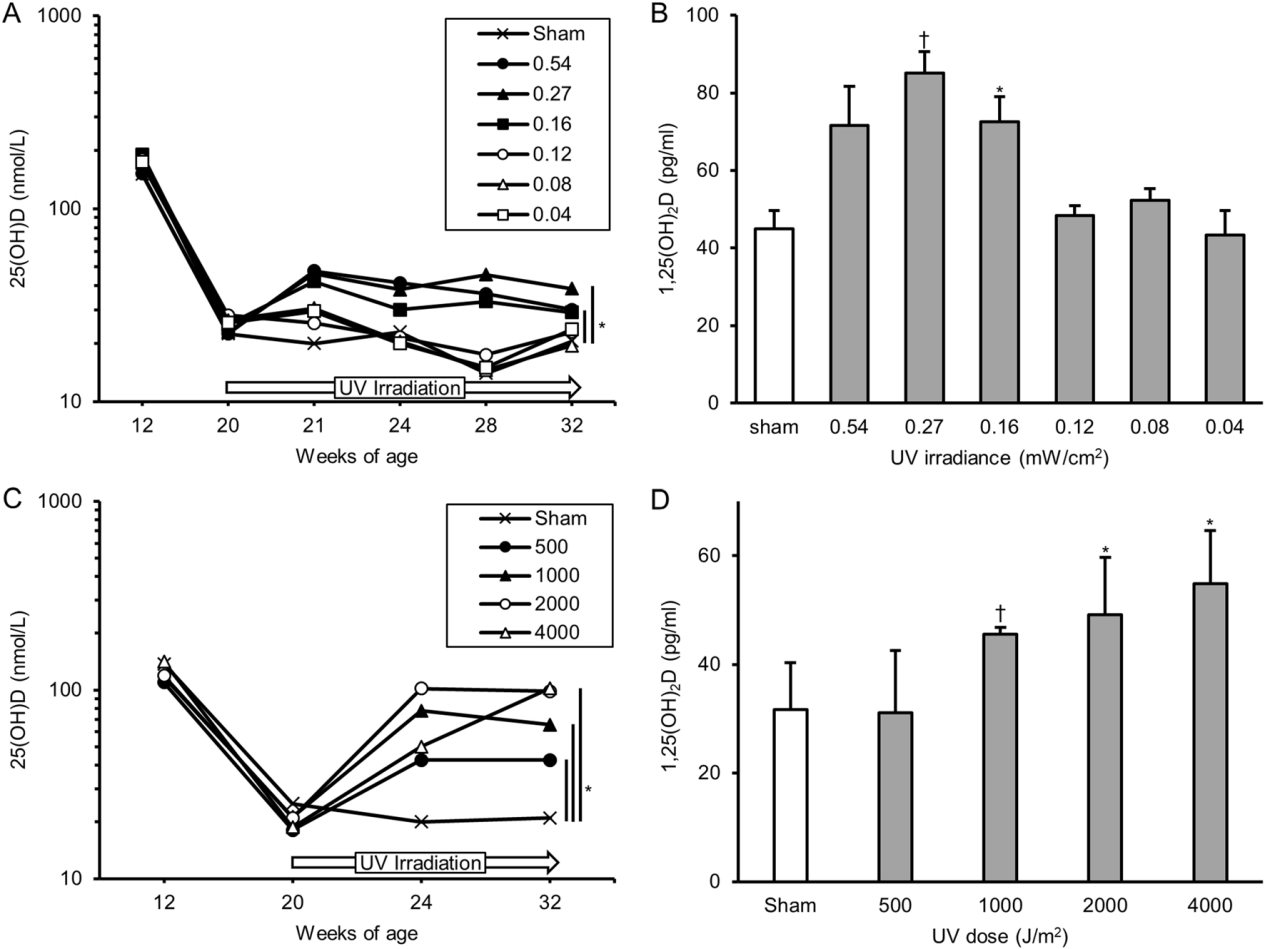
Serum levels of 25(OH)D and 1,25(OH)_2_D in preliminary studies. Serum for 25(OH)D examination was obtained at 12 weeks of age (initiation of vitamin D-deficient diet or vitamin D-containing diet), 20 weeks (initiation of UV irradiation), and 21, 24, 28, 32 weeks. Serum for 1,25(OH)_2_D examination was obtained at 32 weeks (12-weeks of UV irradiation). (**A**) Serum levels of 25(OH)D of irradiance-determination study. (**B**) Serum levels of 1,25(OH)_2_D of irradiance-determination study. (**C**) Serum levels of 25(OH)D of dose-determination study; (**D**) serum levels of 1,25(OH)_2_D of dose-determination study. *p < 0.05. ^†^p < 0.01. *UV* ultraviolet irradiation.

First, we conducted an experiment to determine the minimum irradiance of UV irradiation that adequately supplies vitamin D. It revealed that serum levels of 25(OH)D decreased to a deficiency level (< 25 nmol/L) at 24, 28, and 32 weeks of age in the groups of 0.04, 0.08, 0.12 mW/cm^2^ and sham (Fig. 1A). In the 0.16, 0.27, and 0.54 mW/cm^2^ groups, 25(OH)D levels increased by over 25 nmol/L. Serum levels of 1,25(OH)_2_D at 32 weeks of age were indicated in Fig. 1B.

Next, we conducted an experiment to determine the minimum dose of UV irradiation that adequately supplies vitamin D. Based on the results of the first experiment, the radiant intensity was set to a minimum of 0.16 mW/cm^2^. As indicated in Fig. 1C, serum levels of 25(OH)D decreased to a deficiency level at 24, 32 weeks of age in the sham group. Meanwhile, in the groups with 1,000, 2,000, 4,000 J/m^2^, serum levels of 25(OH)D were maintained at over 25 nmol/L, with this difference significant between the sham mice and all of the other groups (P < 0.001, all groups). Serum levels of 1,25(OH)_2_D at 32 weeks of age were indicated in Fig. 1D. As indicated in Fig. 1D. We considered 0.16 mW/cm^2^ as the minimal UV irradiance and 1,000 J/m^2^ as the minimal dose needed to produce sufficient 25(OH)D in our subsequent main experiments.

### Main experiments

#### Body weight and body composition

At 20 weeks of age, one mouse in the group of vitamin D-repletion without UV irradiation died at the time of blood collection. Finally, we assessed 7 mice in the group of vitamin D-repletion without UV irradiation, and 8 mice in each of the other groups. Gains of body weight were significantly higher in the vitamin D-deficiency − UV irradiation group than those in the vitamin D-deficiency + UV irradiation one (Table 1). However, ratios of fat mass/total mass were significantly increased in the vitamin D-deficiency − UV irradiation group compared with those in the vitamin D-deficiency + UV irradiation one.

**Table 1:** Body weight and ratio of fat mass/total mass and the amounts of change.

	Vit.D+		Vit.D−	
	UV−	UV+	UV−	UV+
**Body weight (g)**				
12 weeks of age	28.2 ± 2.0	29.4 ± 2.0	28.2 ± 1.1	29.8 ± 2.2
36 weeks of age	38.1 ± 7.2	37.9 ± 3.4	38.6 ± 3.3	36.5 ± 3.7
Amount of change	+ 9.9 ± 5.5	+ 8.5 ± 3.0	+ 10.4 ± 3.3*	+ 7.2 ± 2.3
**Fat mass/total mass (%)**				
12 weeks of age	20.5 ± 4.7	21.7 ± 3.7	19.5 ± 2.7	21.2 ± 3.3
36 weeks of age	14.2 ± 3.9	13.5 ± 1.6	14.5 ± 1.5	13.0 ± 2.0
Amount of change	− 6.3 ± 4.7	− 8.2 ± 3.7	− 5.0 ± 2.5*	− 8.2 ± 3.2

*Vit.D−* vitamin D-deficient diet, *Vit.D+* vitamin D-replete diet, *UV* ultraviolet irradiation.

*Significantly different from Vit.D−UV+ group, p < 0.05.

#### Serum metabolites

**Figure 2:**
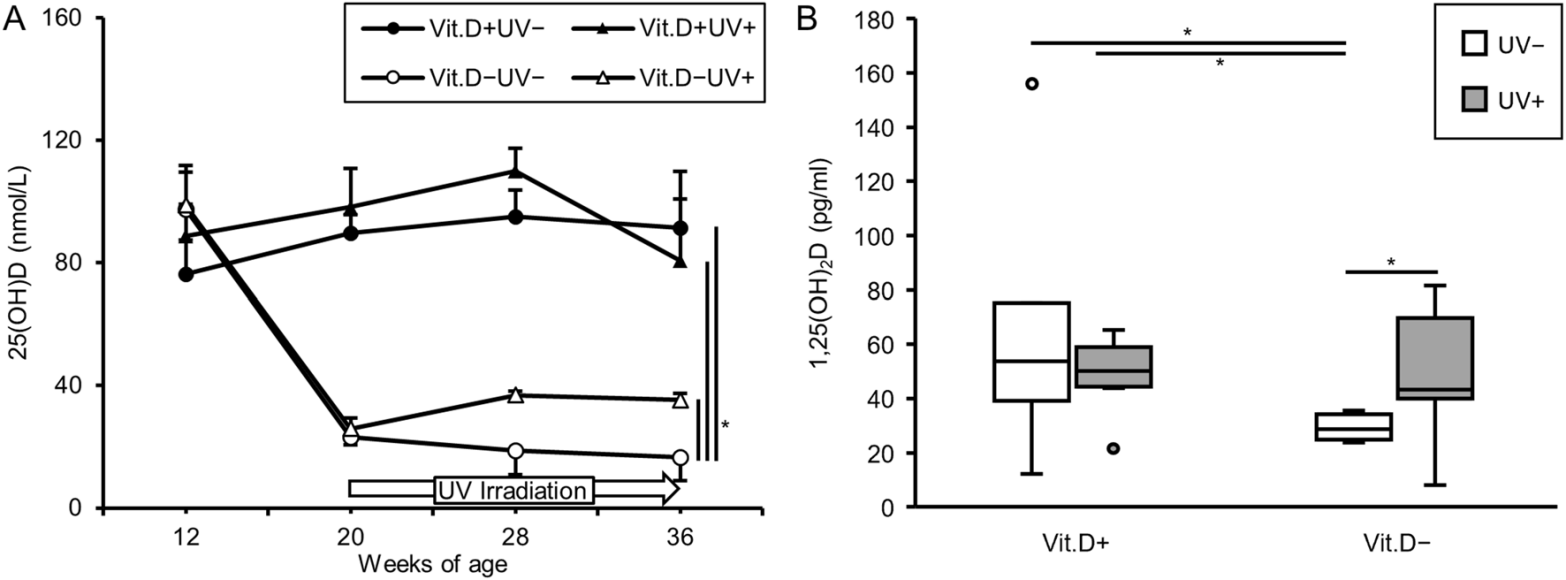
Serum levels of 25(OH)D and 1,25(OH)_2_D in main study. Serum for 25(OH)D examination was obtained at 12 weeks of age (initiation of vitamin D-deficient diet or vitamin D-containing diet), 20 weeks (initiation of UV irradiation), 28 weeks (8-weeks of UV irradiation), 36 weeks (16-weeks of UV irradiation). Serum for 1,25(OH)_2_D examination was obtained at 36 weeks (16-weeks of UV irradiation). (**A**) Serum levels of 25(OH)D. (**B**) Serum levels of 1,25(OH)_2_D. *p < 0.05. *Vit.D−* vitamin D-deficient diet, *Vit.D+* vitamin D-replete diet, *UV* ultraviolet irradiation.

**Figure 3:**
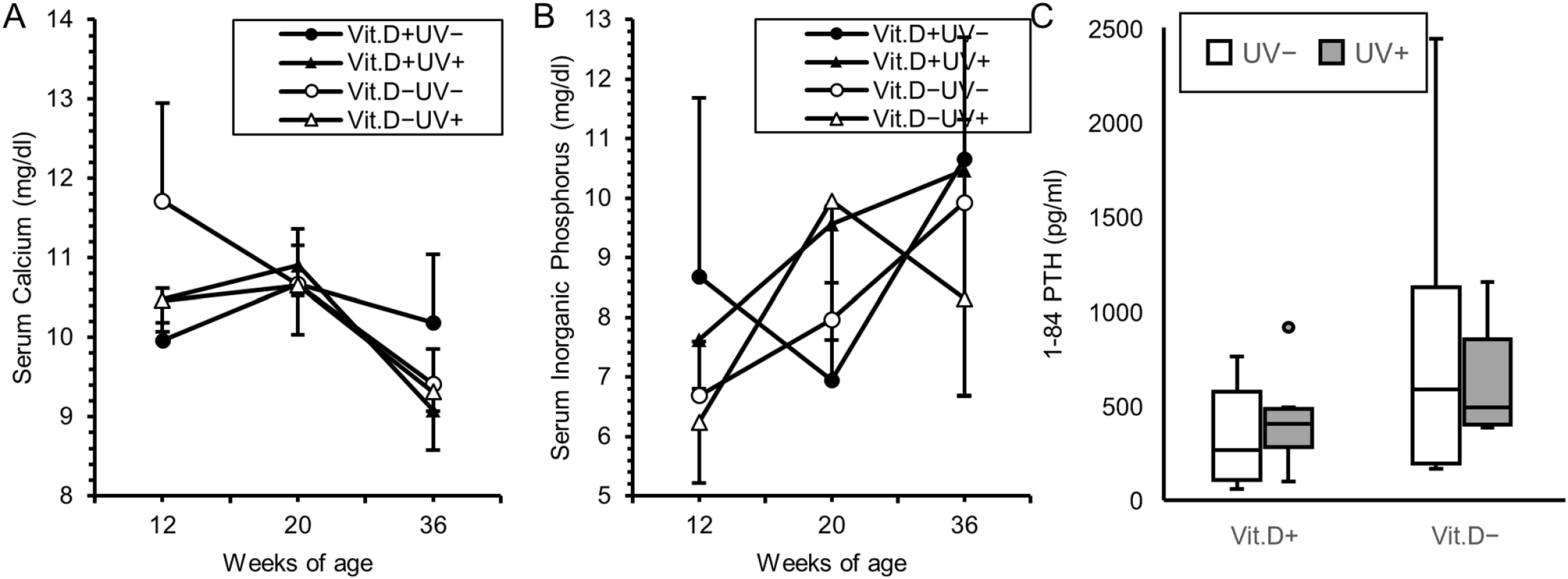
Serum levels of calcium, inorganic phosphorus and 1–84 PTH. Serum for calcium and inorganic phosphorus determination was obtained at 12 weeks of age (initiation of vitamin D–deficient diet or vitamin D-containing diet), 20 weeks (initiation of UV irradiation), 36 weeks (16-weeks of UV irradiation). Serum for 1–84 PTH determination was obtained at 36 weeks (16-weeks of UV irradiation). (**A**) Calcium. (**B**) Inorganic phosphorus. (**C**) 1–84 PTH. *PTH* parathyroid hormone, *Vit.D−* vitamin D-deficient diet, *Vit.D+* vitamin D-replete diet, *UV* ultraviolet irradiation.

From 12 to 20 weeks of age, SAMP6 were fed with the vitamin D-deficient or -replete diet. We examined serum levels of 25(OH)D at 12, 20, 28, 36 weeks of age. As expected, serum levels of 25(OH)D were significantly higher in the groups of vitamin D-repletion than in the vitamin D-deficiency − UV irradiation one. Notably, serum levels of 25(OH)D in the vitamin D deficiency + UV irradiation group were also significantly higher than those in the vitamin D-deficiency − UV irradiation group (Fig. 2A). Serum 1,25(OH)_2_D levels at 36 weeks of age in the vitamin D-deficiency + UV irradiation group were also significantly higher than in the vitamin D-deficiency − UV irradiation one (Fig. 2B). There were no differences in serum levels of Ca or IP at 12, 20, or 36 weeks of age among the four groups (Fig. 3A,B). As indicated in Fig. 3C, serum levels of PTH in the vitamin D-deficiency − UV irradiation group were higher than those in the other groups. However, there was no significant difference.

#### Real-time RT-PCR analysis

**Figure 4:**
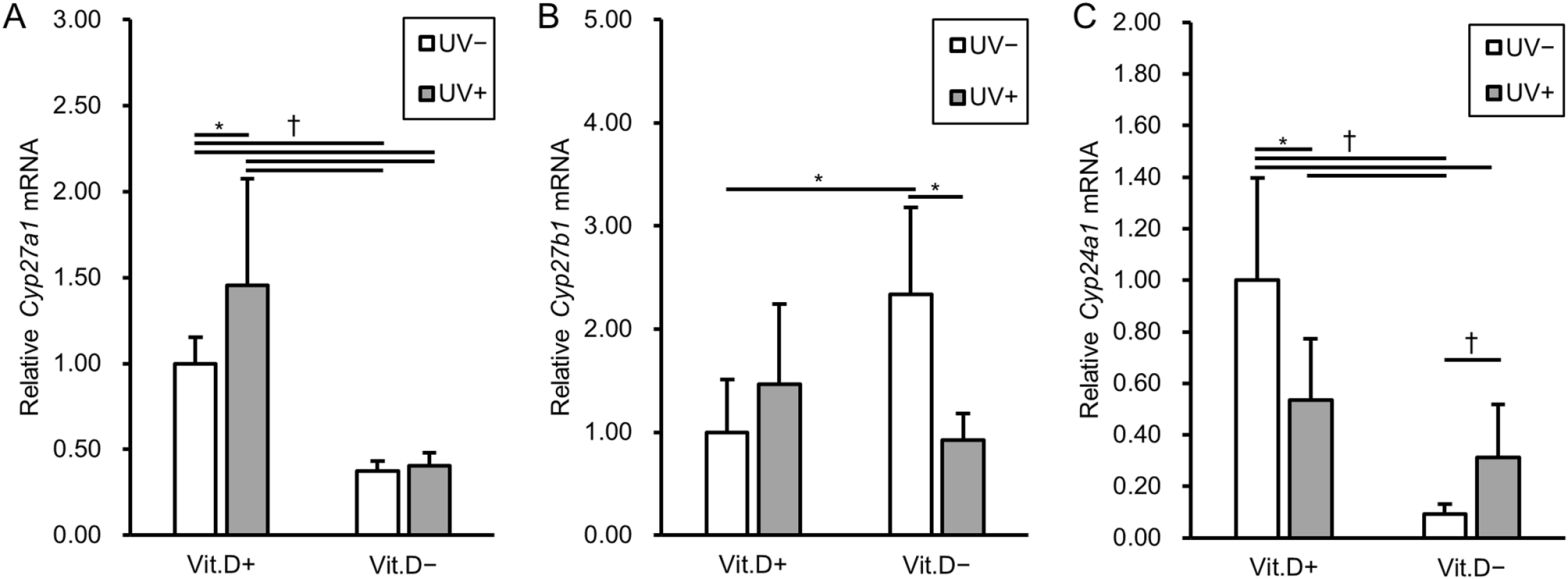
Levels of mRNA expression in association with vitamin D metabolism. Relative expression levels in each group are expressed with reference to that in Vit.D+UV− group as 1.0. Levels of all target mRNA were normalized with those of *Gapdh* mRNA. (**A**) Relative *Cyp27a1* mRNAs. (**B**) Relative *Cyp27b1* mRNAs. (**C**) Relative *Cyp24a1* mRNAs. *p < 0.05. *Vit.D−* vitamin D-deficient diet, *Vit.D+* vitamin D-replete diet, *UV* ultraviolet irradiation.

We investigated levels of mRNA expression for one of C25-hydroxylases (*Cyp27a1*, liver sample), 25 hydroxyvitamin D-1-alpha hydroxylase (*Cyp27b1*, kidney sample), and 1,25-dihydroxyvitamin D 24-hydroxylase (*Cyp24a1*, kidney sample) by real-time RT-PCR. As indicated in Fig. 4A, the mRNA levels of *Cyp27a1*, which is responsible for the conversion of vitamin D into the stored form, 25(OH)D, were significantly higher in the vitamin D-replete groups than -deficient groups. The mRNA levels of *Cyp27b1*, which is responsible for the conversion of 25(OH)D into the active form 1,25(OH)_2_D in the kidney, were significantly higher in the vitamin D-deficiency − UV irradiation group than in the groups of vitamin D-repletion − UV irradiation or vitamin D-deficiency + UV irradiation (Fig. 4B). In contrast, the mRNA levels of *Cyp24a1*, which is responsible for the conversion of active 1,25(OH)_2_D into the inactive form, were significantly lower in the vitamin D-deficiency − UV irradiation group than in the other groups (Fig. 4C). The results of mRNA expression levels for enzymes may reflect the facts that 25(OH)D and 1,25(OH)_2_D levels were insufficient in the group of vitamin D-deficiency − UV irradiation, but higher in the vitamin D-deficiency + UV irradiation ones. Whereas there was no significant difference in levels of mRNA expression for *Cyp2r1* (liver sample) (Suppl. Figure 1).

#### Analyses of bone morphology with micro-CT measurement

**Figure 5:**
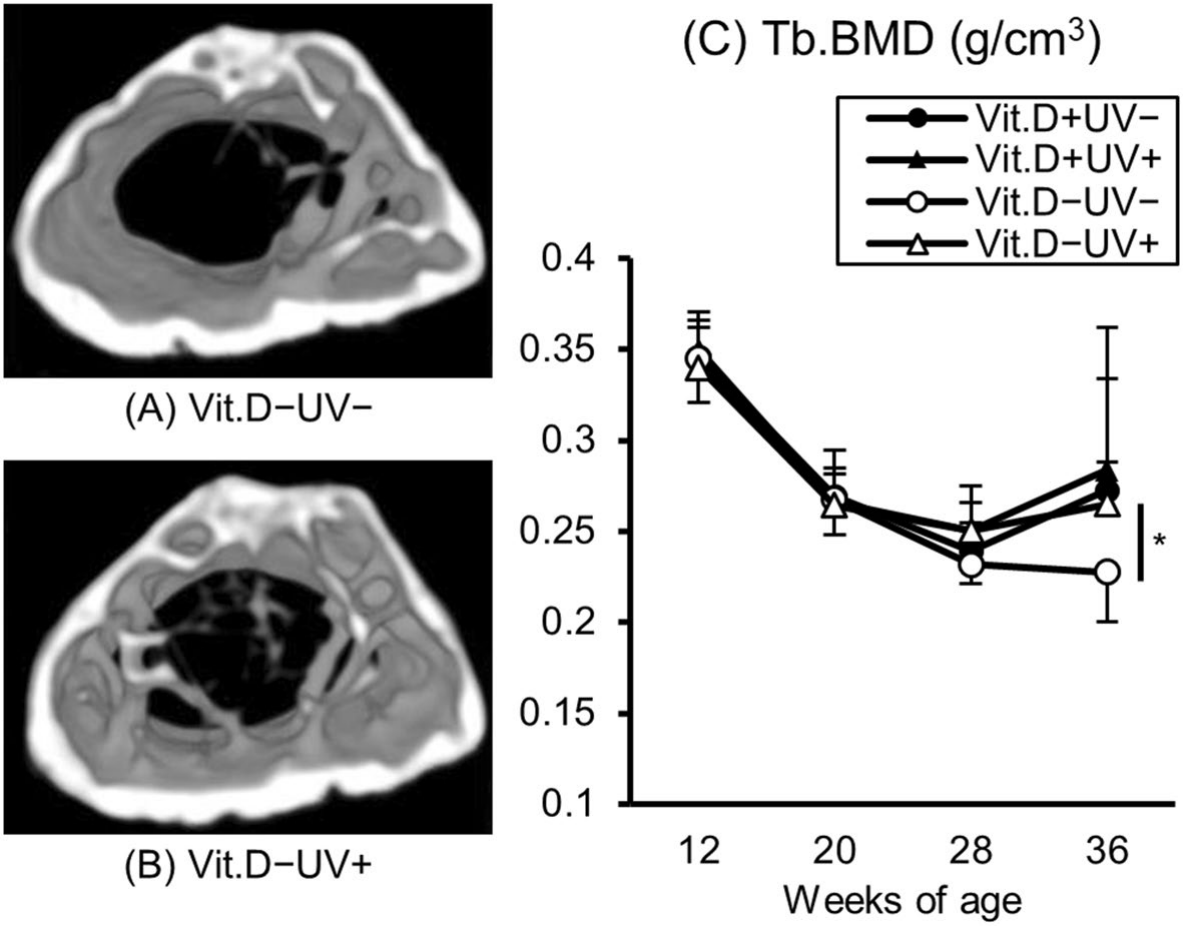
Three–dimensional digital images and Tb.BMD of right femur. Three-dimensional images of right femur were reconstructed and Tb.BMD were calculated based on micro–CT data at 36 weeks of age. (**A**) Vit.D−UV− image from a distal viewpoint. (**B**) Vit.D−UV+ image from a distal viewpoint. (**C**) Tb.BMD of right femur. Vit.D−UV+ group showed higher density of trabecular bone than that in Vit.D−UV− group. *p < 0.05. *Vit.D−* vitamin D-deficient diet, *Vit.D+* vitamin D-replete diet, *UV* ultraviolet irradiation, *Tb.BMD* trabecular bone mineral density.

Results of bone morphology analyses with micro-CT measurement were shown in Table 2. Notable results were that the Tb.BMD was significantly higher in the vitamin D-deficiency + irradiation (265 ± 23 g/cm^3^) than vitamin D-deficiency − irradiation (227 ± 27 g/cm^3^) group. Actual CT images revealed thickened trabecular bone in the vitamin D-deficiency + UV irradiation as compared with the vitamin D-deficiency − UV irradiation group (Fig. 5A,B). After the irradiation, a temporary increase of Tb.BMD was observed in the vitamin D-deficiency + UV irradiation group (Fig. 5C). No significant improvements in any other trabecular or cortical bone parameters were noted in the vitamin D-deficiency + UV irradiation groups as compared with the vitamin D-deficiency − UV irradiation group.

**Table 2:** Bone morphology with micro-CT measurement at 36 weeks of age.

	Vit.D+		Vit.D−	
	UV−	UV+	UV−	UV+
Tb.BV/TV (%)	25.6 ± 9.5	24.2 ± 6.8	19.2 ± 7.1	18.6 ± 3.0
Tb.Th (µm)	83.0 ± 23.5	80.6 ± 16.0	71.5 ± 23.6	65.9 ± 8.2
Tb.N (1/mm)	3.03 ± 0.33	2.98 ± 0.51	2.67 ± 0.32	2.84 ± 0.42
Tb.Sp (µm)	348 ± 82	345 ± 82	409 ± 73	393 ± 50
Tb.BMD (mg/cm^3^)	273 ± 61	284 ± 79	227 ± 27*	265 ± 23
Ct.Th (µm)	248 ± 17	245 ± 14	246 ± 16	240 ± 11

*CT* computed tomography, *Vit.D−* vitamin D-deficient diet, *Vit.D+* vitamin D-replete diet, *UV* ultraviolet irradiation, *BV* bone volume, *TV* tissue volume, *Tb.BV/TV* trabecular percent bone volume, *Tb.Th* trabecular thickness, *Tb.N* trabecular number, *Tb.Sp* trabecular separation, *Tb.BMD* trabecular bone mineral density, *Ct.Th* cortical thickness.

*Significantly different from Vit.D−UV+ group, p < 0.05.

#### Mechanical test

**Figure 6:**
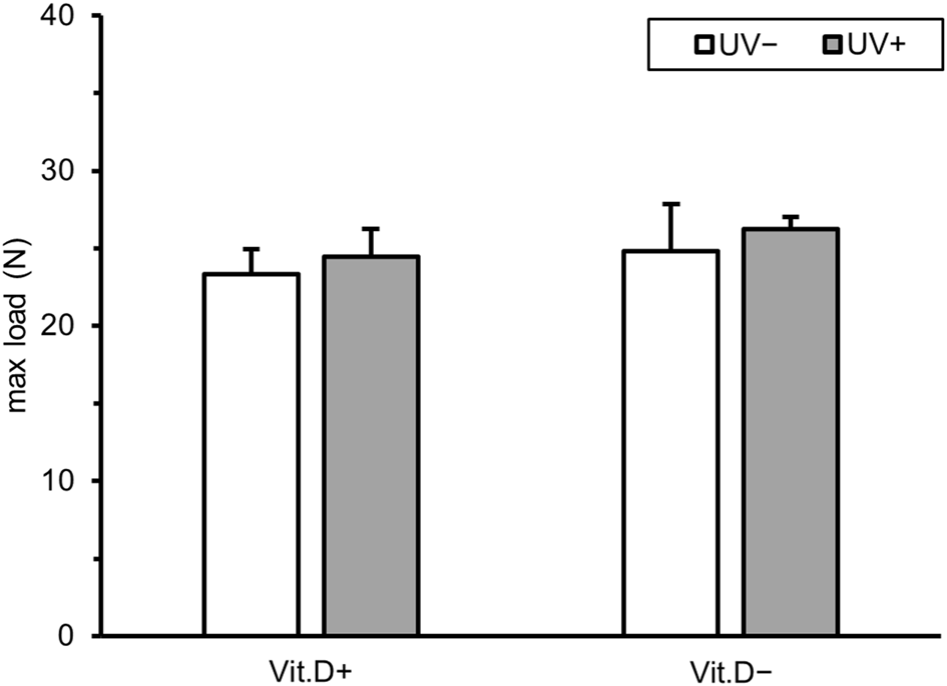
Results of mechanical test. Results of three point bending test of left femur at 36 weeks of age are graphed. *Vit.D−* vitamin D-deficient diet, *Vit.D+* vitamin D-replete diet, *UV* ultraviolet irradiation, *N* newton.

There was no difference in mechanical strength among the four groups determined by the 3-point bending test (Fig. 6).

#### Bone histology

**Figure 7:**
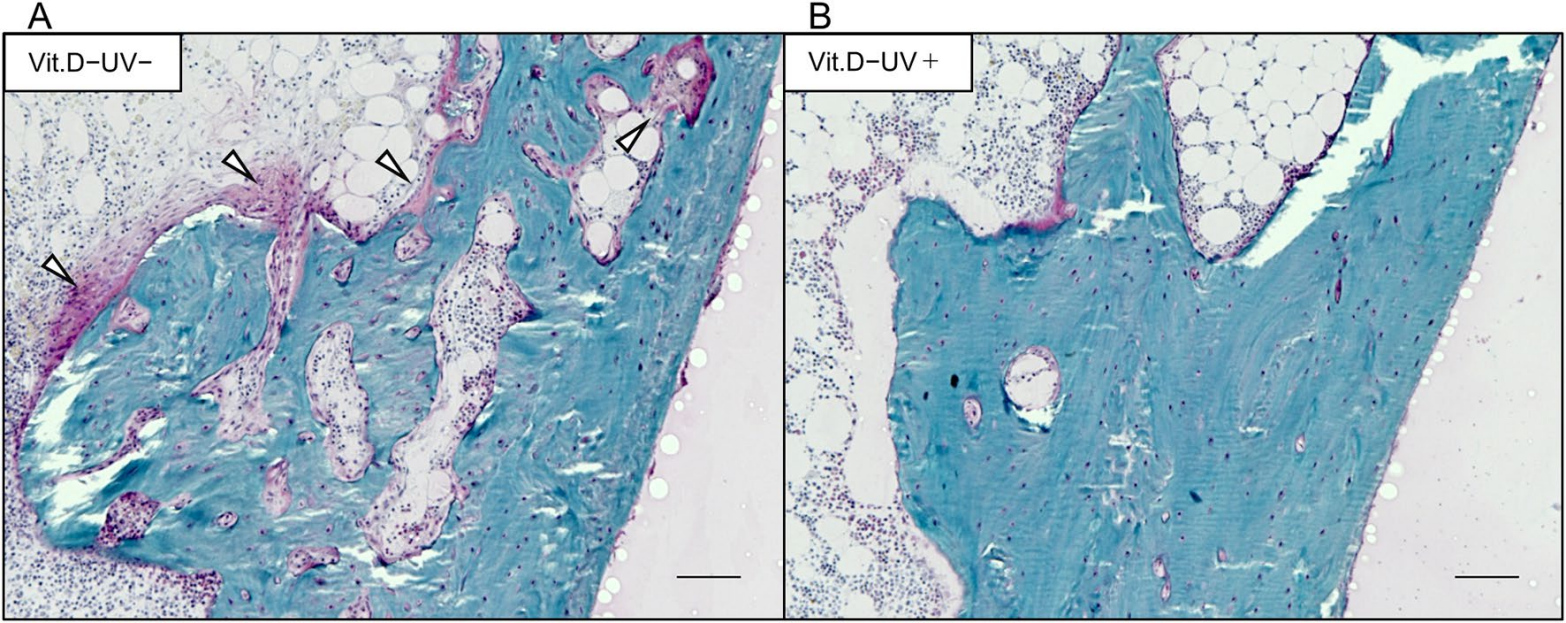
Results of Villanueva Goldner staining. Coronal sections of the medial metaphysis of left femurs at 36 weeks of age were stained (original magnification × 200, bars indicate 100 μm). (**A**) Vit.D−UV− group. (**B**) Vit.D−UV+ group. A greater amount of red-colored osteoid tissues (white arrowheads) was observed in Vit.D−UV− group than in Vit.D−UV+ group. *Vit.D−* vitamin D-deficient diet, *Vit.D+* vitamin D-replete diet, *UV* ultraviolet irradiation.

On Villanueva Goldner staining the amount of red-colored osteoid tissues, representing incomplete calcification of the bone matrix, was greater in the vitamin D-deficiency − UV irradiation group than in those with UV irradiation (Fig. 7).

**Figure 8:**
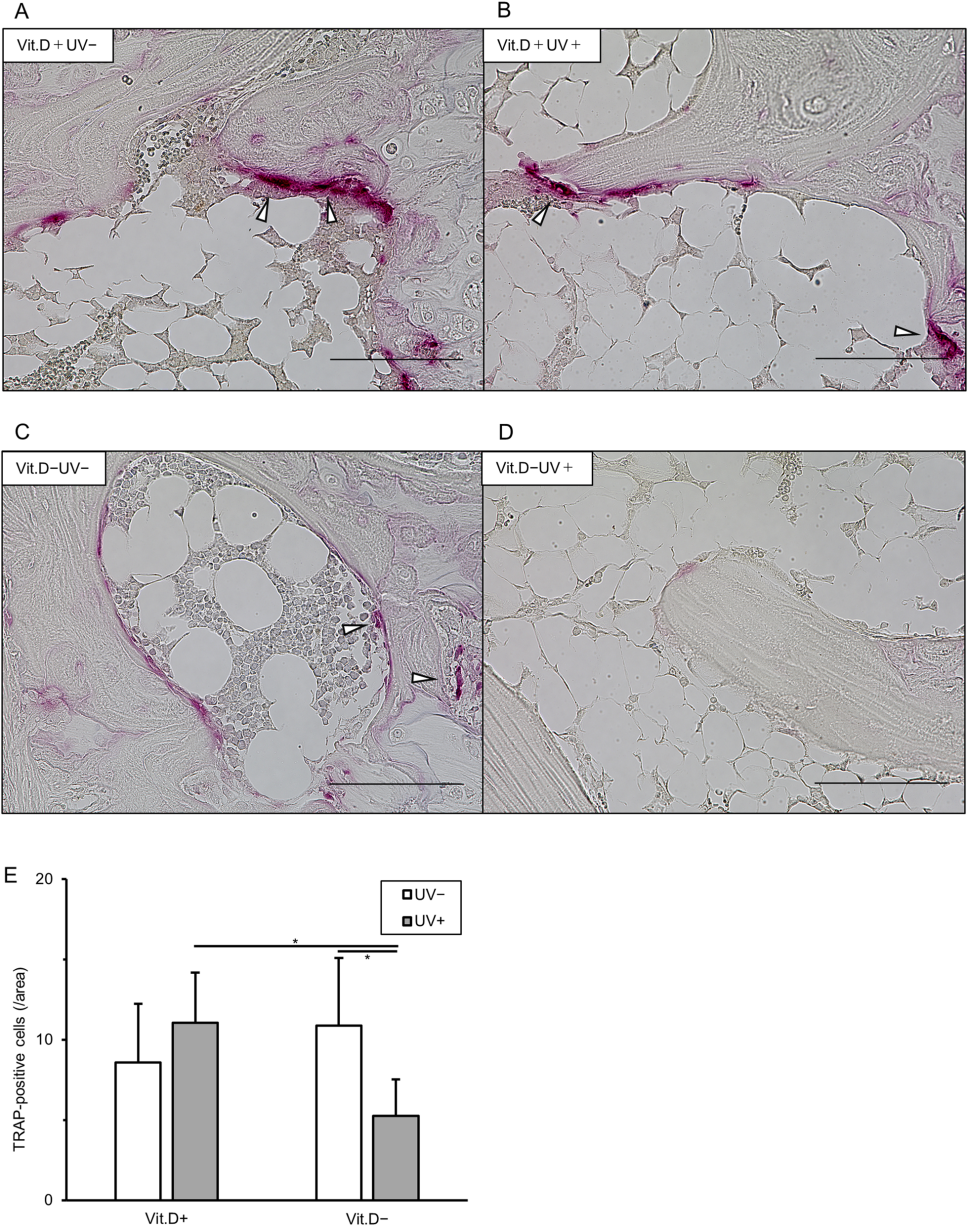
Results of TRAP staining. (**A**)–(**D)** For sagittal sections of proximal metaphysis of the left tibia were stained with TRAP original magnification × 400, bars indicate 100 μm). White arrowheads show osteoclasts stained with TRAP. (**E**) Quantification of TRAP-positive cell numbers (/area, white arrowheads) in randomly selected 5 low-power fields (× 100). *p < 0.05. *TRAP* tartrate-resistant acid phosphatase, *Vit.D−* vitamin D-deficient diet, *Vit.D+* vitamin D-replete diet, *UV* ultraviolet irradiation.

By quantitative analysis, osteoclasts (/area) tended to be fewer in the vitamin D-deficiency + UV irradiation compared with vitamin D-deficiency − UV irradiation group (Fig. 8).

#### Grip strength, muscle mass, muscle histology

**Figure 9:**
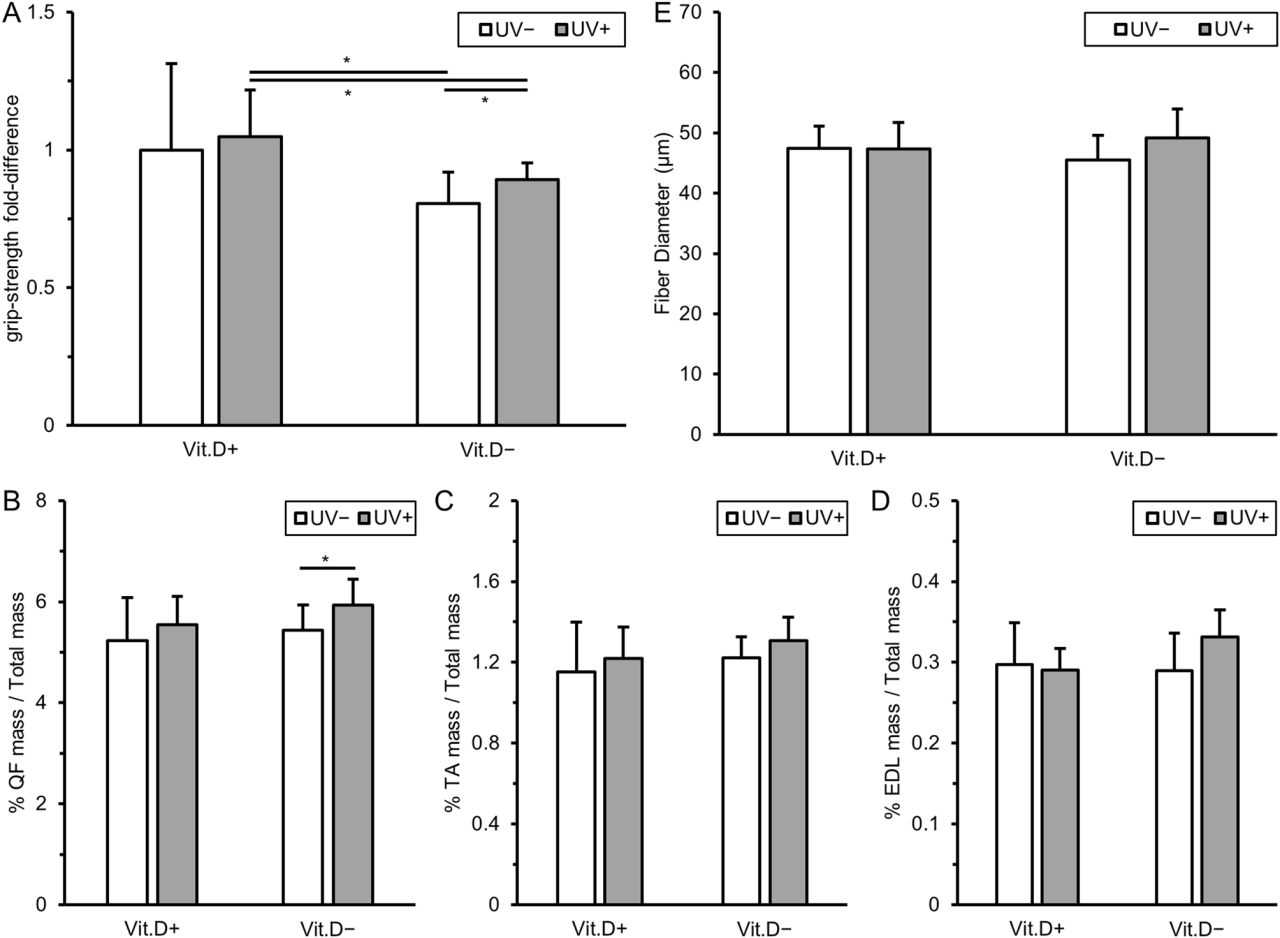
Results of muscle strength and muscle mass evaluation. (**A**) Grip strength at 36 weeks of age was expressed with reference to that in Vit.D+UV− group as 1.0. measured in 15 trials per mouse. The values were corrected for body weight. (**B**)–(**D**) Muscle weight at 36 weeks of age corrected by body weight. (**E**) Fiber diameters of tibialis anterior. Fiber diameters were averaged by 30 fibers randomly selected per mouse. *p < 0.05. *Vit.D−* vitamin D-deficient diet, *Vit.D+* vitamin D-replete diet, *UV* ultraviolet irradiation, *QF* quadriceps femoris, *TA* tibialis anterior, *EDL* extensor digitorum longus.

As indicated in Fig. 9A, there were differences in grip strength between the vitamin D-replete group + UV irradiation versus -deficient groups. More interestingly, grip strength was higher in vitamin D-deficiency + UV irradiation than in vitamin D-deficiency − UV irradiation, indicating that UV irradiation increases the grip strength in the presence of vitamin D-deficiency. Regarding muscle mass, vitamin D-deficient mice with UV irradiation had significantly heavier quadriceps muscles after adjustment for total body mass compared to vitamin D-deficient ones without irradiation (Fig. 9B). Increased muscle mass was also observed in TA and EDL muscles, although the differences were not significant (Fig. 9C,D). Vitamin D-deficiency + UV irradiation mice displayed larger quadriceps muscle fibers than vitamin D-deficiency − UV irradiation ones (p = 0.105) (Fig. 9E).

#### Skin histology

**Figure 10:**
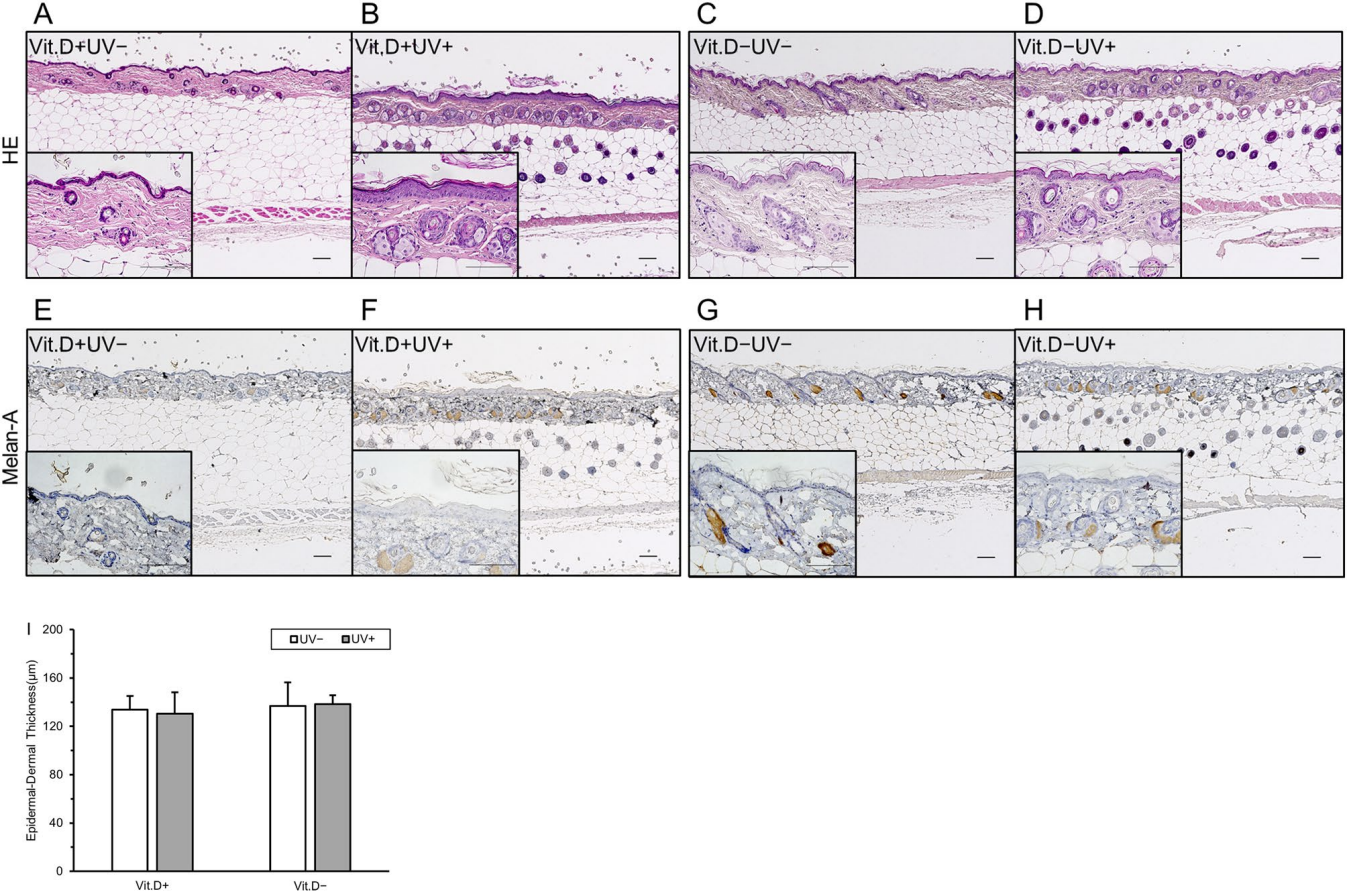
Influence of UV irradiation on skin tissues. (**A**)–(**D**) Hematoxylin and eosin staining for epidermis and dermis, (original magnification × 10, bars indicate 100 μm). (**E**)–(**H**) Immunohistochemical staining with anti-Melan-A monoclonal antibody for epidermis and dermis (original magnification × 100, bars indicate 100 μm). (**I**) Quantification of epidermal and dermal thickness in 10 randomly selected area of low-power fields (× 100). *Vit.D−* vitamin D-deficient diet, *Vit.D+* vitamin D-replete diet, *UV* ultraviolet irradiation, *HE* hematoxylin and eosin staining, *Melan* immunohistochemical staining with anti-Melan-A monoclonal antibody.

An important issue addressed in the present study is the need to reduce the side effects induced by irradiation. The most notable side effects occur in the skin. Minor reorganization was observed in the skin tissues of the UV-irradiated group with HE staining (Fig. 10A–D), however, no signs of gross damage. In quantitative analysis, there were no differences in the skin epidermal or dermal thicknesses among the groups (Fig. 10I). As for immunohistochemistry, there were no melanocytes or keratinocytes with melanin pigmentation in the epidermal areas in any group (Fig. 10E–H). Immunostaining of cleaved caspase 3 and TdT-mediated dUTP nick end labeling (TUNEL) were shown in Supple. Figure 2, and histology and immunostaining of damaged skin by shorter wave-length UV (305 nm) irradiation were shown in Suppl. Figure 3. There seemed to be difference in positive stainability of cleaved caspase 3 and TUNEL staining between skins with and without UV irradiation.

## Discussion

This is the first report demonstrating that low energy narrow-range UV irradiation from a UV-LED device is effective in improving osteoporotic and sarcopenic changes in the presence of vitamin D deficiency in a senescence-accelerated mouse model. We documented that low energy and narrow-range UV irradiation improved bone mineral density, muscle volume and strength in the vitamin D deficient group. Furthermore, low energy and narrow range (316 nm) UV irradiation did not induce severe skin damages, suggesting that the equipment used may be safe for osteosarcopenia therapy.

Our previous study showed that UV irradiation by 305 nm LED, which is UV-B region, improved the morphological characteristics and mechanical strength of bone as well as histological features in a vitamin D deficient senile osteoporotic mouse model^12^. Because of the irradiation with lower energy intentionally used in the present study as compared with the 305 nm UV irradiation in our previous study, the increase of serum levels of 25(OH)D was less marked than in our previous study. This would explain why there was no difference in the mechanical strength of femur between the irradiation + and irradiation − groups in the present study. But bones in the vitamin D-deficiency + UV irradiation group showed partly better bone morphology and micro CT and histological features than those without UV irradiation. The most important aim in this study was to increase levels of 25(OH)D by administering low energy UV irradiation with minor side effects. Improvement of mechanical strength would be obtained by irradiation with the current low-energy LED for a longer period.

Several previous reports documented the efficacy of UV-irradiation and 1,25(OH)_2_D uptake for osteoporotic condition. Guo et al.^15^ reported that long-term UV irradiation (12 weeks) improved bone mineral content and bone mineral density in young rats, although they used wavelengths of 280–340 nm with the wave peak at 315 nm. In the present study, we used a narrow range wavelength of 305–325 nm with the wave peak at 316 nm, which reduces the risk of side effects by LED irradiation, particularly with a longer duration of irradiation.

Geldenhuys et al.^16^ reported that body weight and percentage of fat mass were decreased by UV irradiation in a murine study. Similarly, mice in the vitamin D-deficiency + UV irradiation group showed a decrease in body weight greater than that of the vitamin D-deficiency − UV irradiation group in the present study. Despite the lower body weight induced by UV irradiation, the bone mineral density in the vitamin D-deficiency + UV irradiation group was improved and bone strength was maintained, which is a noteworthy result for this LED experiment. Duque et al.^17^ reported that short-term infusion (6 weeks) of 1,25(OH)_2_D was effective for preventing osteoporosis, particularly for periosteal ossification and BMD in SAMP6 mice, which is the same mouse model used in the present study. In SAMP6, ovariectomy does not increase osteoclast activity or reduce bone mass^18^. This mouse strain is characterized by reduced osteoblast formation and increased adipogenesis in the bone marrow, causing bone loss and fracture and mimicking the human form of senile osteoporosis. This model might be adequate for analyzing the efficacy of LED equipment in the treatment of osteoporosis in the super-aging society of developed countries. However, the experiments conducted in Duque’s study cannot be repeated in the clinical setting because of the infusion of 1,25(OH)_2_D.

Some in vivo studies reported that the active form of vitamin D decreases the pool of osteoclast precursor cells^19^, and blocks osteoclastic bone resorption by mobilizing precursor monocytes from the bone to the blood^20^. Our histological assay showed inhibitory effects of increased active form of vitamin D by LED irradiation on TRAP positive osteoclast expression in vivo, which was consistent with the results of these previous reports. Interestingly, although levels of serum Ca and IP did not decrease in the vitamin D-deficiency mouse group, levels of serum PTH tended to increase (not significant). Experiments with accumulating number of mice will more clarify that secondary hyperparathyroidism possibly caused by vitamin D-deficiency could influence the bone turnover in vitamin D-deficient mice. Thus, the active form of vitamin D induced by UV-LED might prevent osteoclastic bone loss.

Together with osteoporosis treatment, sarcopenia treatment or prevention is also important in the elderly. Several studies demonstrated impaired muscle contraction and decrease of grip strength in vitamin D-deficient rats and mice^13,21^. In our study, vitamin D-deficiency reduced grip strength, while UV irradiation improved it significantly. Muscles from vitamin D-deficient mice were significantly lighter and UV irradiation increased muscle mass. These results might have been influenced by the body weight decrease seen in the vitamin D-deficiency group, with in particular this decrease being greater with than without UV irradiation. Body weight and body fat percentage in vitamin D-deficiency + UV irradiation were decreased as previously reported^16^. Together, UV irradiation increased muscle volume, while decreasing body weight and percentage of body fat, which might be positive changes for sarcopenia therapy. Girgis et al. showed that muscle mass from vitamin D-deficient mice tends to be light, but there was no significant difference in their study using C57BL/6 mice. The reason for the larger difference in the present study may be attributable to the fact that the periods of both the vitamin D-deficient diet and UV irradiation were longer in it than in Girgis’s study. Another reason might be that we used senescence-accelerated mice in the present studies, which would be a more suitable mouse model of the human form of senile sarcopenia^22,23^.

The most important aim of the present study is to provide therapeutic UV at wavelengths with few side effects. In our previous study, UV irradiation by 305 nm thickened epidermis and dermis, and also generated few Melan-A positive cells. In contrast, in the present study, low energy UV irradiation by 316 nm irradiation did not induce such signs of skin damage. Considering the UV-index, the previous irradiance with 305 nm of UV was equivalent to 6.75 points of the UV index based on WHO criteria, which was defined as high risk. In the present study, the irradiance with 316 nm UV was equivalent to 2.00 points, which was defined as safe. Because osteosarcopenia can be efficiently and safely improved under the conditions set in the present study, it can be applied to human clinical practice.

There were several limitations in this study. First, we measured serum vitamin D levels not by liquid chromatograph tandem mass spectrometry (LC–MS/MS) but by radioimmunoassay. LC–MS/MS would be better to measure the exact amount of vitamin D in individual patients, while radioimmunoassay would be adequate to compare the serum vitamin D levels of mice in the same environment. Second, we measured mRNA of vitamin-D metabolic enzymes, bone strength, histology and serum 1,25(OH)_2_D at the end of the irradiation period. Collecting data during the irradiation period would be preferable for explanation of the interim progression, but would need the further sacrifice of numerous mice. In addition, frequent tests for serum 1,25(OH)_2_D levels require large amounts of blood, which can cause unexpected death in mice. To prove the effects of UV irradiation on improvement of osteosarcopenia, we set minimum required evaluation points in the present study. Third, in preliminary experiments, results of serum 25(OH)D level were different between irradiance-determination and dose-determination experiments. Possible explanation was that the time when the experiment was conducted was different, and the numbers of mice were small in preliminary experiments. Fourth, we set preliminary and main experiments in the present study, in each of which the mice were different. Results of serum 25(OH)D levels were slightly different between the mice (preliminary and main experiments). However, the effective irradiance and dose determined by the preliminary experiments could also improve osteosarcopenia in the main experiments. Fifth, the scale of serum 25(OH)D level used in the present experiments were not applicable directly to human. In addition, we should examine the effects of UV irradiation also on the male mice because of the proportion of osteoporotic fractures in men cannot be ignored and is a crucial problem in an aging society.

In conclusion, our results indicated that conditions of low energy UV irradiation by 316 nm LED device determined by preliminary experiments improved osteosarcopenia in a vitamin D deficiency senile mice model. UV irradiation with this UV-LED device could be clinically useful for patients with osteosarcopenia with few side effects, particularly, patients with low mobility, and/or cannot sunbathe. Since this device could be developed as a small and portable one, it could be easy to use in a variety of situations in the clinical setting from a general hospital to home-care. Considering that many developed countries face an increasingly aged population that is more susceptible to the burdens of osteosarcopenia associated with vitamin D deficiency, treatment by low energy UV irradiation with a narrow range UV-LED device may be a promising novel therapeutic approach to this disease.

## Materials and methods

### Study design: preliminary and main experiments

**Figure 11:**
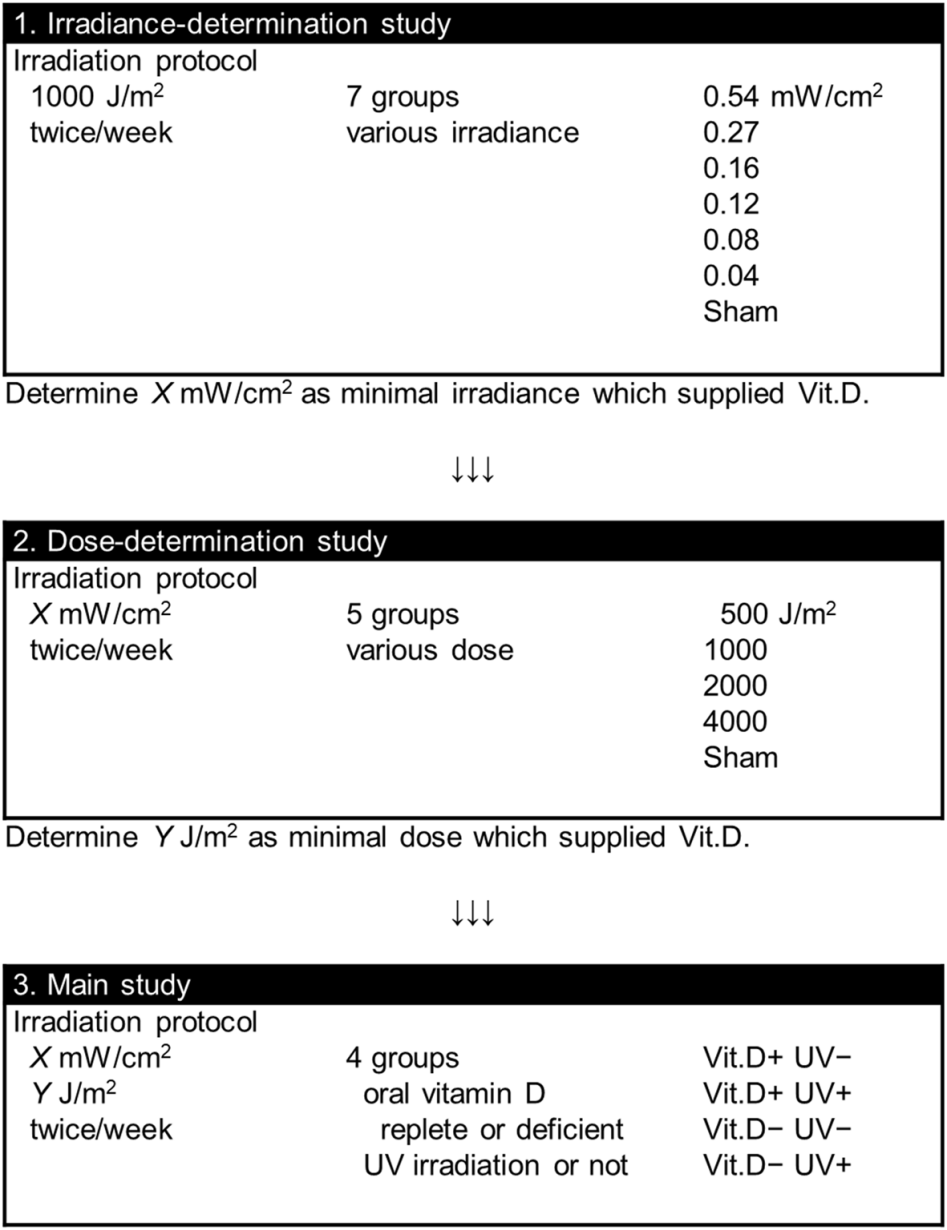
Flowchart of preliminary and main experiments.

All experimental procedures on animals were approved by the Institutional Animal Utilization Study Committee of Nagoya University (Permit Number; 28106), and were implemented in accordance with the National Institutes of Health Guide for the Care and Use of Laboratory Animals. All experiments were performed under isoflurane anesthesia, and all efforts were made to minimize the suffering of the mice used in the experiments. As a prerequisite for developing treatment equipment with low-cost and high safety, we planned to determine the lowest irradiance and dose of UV-LED that would be effective to supply sufficient levels of serum vitamin D as preliminary experiments. Using the determined irradiance and dose of UV-LED, the effects of this condition were evaluated on the osteoporosis and sarcopenia mimicking models of SAMP6 as the main experiments. A flowchart of the present experimental design can be seen in Fig. 11.

### Preliminary experiments

#### Mice and diet

Inbred C57BL/6JJ female mice were purchased from Japan SLC, Inc. (Hamamatsu, Japan). Because a previous study indicated that female mice were more sensitive than male mice to vitamin D supplementation by UV irradiation^24^, we used only female mice in the present study. They were kept at a temperature of 25 °C with a 12-h light–dark cycle and shielded from UVB of an ordinary fluorescent light. To create a 25-hydroxyvitamin D [25(OH)D]-starved mouse group for the experiments, mice were weaned from their mothers and fed the standard wheat-based mouse diet until 12 weeks of age. Then they were fed a vitamin D-deficient diet (AIN93GA-2, Oriental Yeast Co ltd., Tokyo, Japan) until the study termination^25^, at 32 weeks of age. AIN93GA-2 contains no vitamin D, 0.50% calcium, 0.20% phosphorus, and 7.00% total fat.

#### UV irradiation

Surface-mounted device-packaged UV lamps of the LED system developed by Nikkiso Co., ltd. (Tokyo, Japan) in collaboration with Dr. Hiroshi Amano in our institution were used as the UV source. To ascertain the effective minimal UV intensity in detail, we used seven different irradiance intensities: 0.04, 0.08, 0.12, 0.16, 0.27, 0.54 mW/cm^2^ and a control group. The highest irradiance (0.54 mW/cm^2^) had been used in our previous work^11,12^, and the middle intensity (0.16 mW/cm^2^) was considered as a “safe” intensity, equivalent to a UV index of 2.00^26^. We adjusted the LED module to a wavelength of 316 nm, because 316 nm was already confirmed to effectively provide vitamin D, and considered to be less harmful owing to its being at the short end of UVA^26^. The wave spectrum of the LED module was measured with a UV radiometer USR-45 DA-10 (Ushio Inc., Tokyo, Japan), and the spectrum was proved to be within a very narrow range (Suppl. Figure 4). As previously described, a 2 × 4 cm dorsal patch of skin was clean-shaven as the area to be irradiated^27^. We irradiated the mice in a clear acrylic box with a base area of 4 × 6 cm. The lamp was positioned 10 cm above the dorsal patch of the mice. The radiation irradiance on the area of the dorsal patch in the box for the LED module was measured using a UV radiometer USR-45 DA-10 (Ushio Inc., Tokyo, Japan). The reflection coefficient of the box was 1.77. The UV irradiation dose was controlled to 1,000 J/m^2^. At 20 weeks of age, forty mice were divided into 7 groups (5 mice each); a control group and six UV irradiation groups (0.04, 0.08, 0.12, 0.16, 0.27, 0.54 mW/cm^2^, 1 kJ/m^2^, 316 nm, irradiated twice a week) (Fig. 11). We allocated mice to experimental groups by matching the serum 25(OH)D level and body weight. Irradiation time was adjusted to 185–624 s to make the dose equal to 1,000 J/m^2^ at each irradiance. They were fed a vitamin D deficient diet from 12 weeks of age, and irradiated with UV from 20 to 32 weeks of age according to a previous study^12^. At the age of 32 weeks, the mice were sacrificed, and subjected to serum analyses.

After the irradiance-determination study, we similarly performed a dose-determination study. At 20 weeks of age, the mice were divided into five groups (4 mice each; a control group and four UV irradiation groups (500, 1,000, 2,000, 4,000 J/m^2^, 316 nm, irradiated twice a week) (Fig. 11). We divided mice by matching the serum 25(OH)D level and body weight. The irradiance intensity used in this dose-determination study was set at a minimal value determined by the irradiance-determination study, which supplied sufficient 25(OH)D. They were fed a vitamin D deficient diet from 8 weeks of age, and irradiated with UV from 20 to 32 weeks of age. At the age of 32 weeks, the mice were sacrificed, and subjected to serum analysis.

#### Serum metabolites

Serum 25(OH)D levels were measured at 12 weeks of age (pre-diet), 20 weeks (pre-UV irradiation), and 24, 28, 32 weeks (4, 8, 12 weeks’ UV irradiation), and 1,25(OH)_2_D levels at 32 weeks. Each level was measured with radioimmunoassay kits (SRL, Tokyo, Japan) following the manufacturer's protocol. Blood samples were obtained from the plexus of the orbital vein, and stored at − 20 °C until quantification. The lower limit of quantification for serum 25(OH)D was 12.5 nmol/L. The vitamin D levels were classified as follows: deficiency, 25(OH)D < 25 nmol/L; sufficiency, 25(OH)D > 90 nmol/L, as described previously^28^.

### Main experiments

#### Mice and diet

SAMP6 were purchased from Japan SLC, Inc. (Hamamatsu, Japan) after approval was obtained from the Council for SAM Research (Kyoto, Japan). We used only female mice also for the preliminary experiments. Then they were fed a vitamin D-deficient diet or a vitamin D-replete diet (AIN93GA-2, Oriental Yeast Co ltd., Tokyo, Japan) from 12 weeks of age until the study termination.

#### Treatment groups and UV irradiation

Thirty-two mice were divided into 4 groups: oral vitamin D-repletion without UV irradiation, oral vitamin D-repletion with UV irradiation, vitamin D-deficiency without UV irradiation, and oral vitamin D-deficiency with UV irradiation (Fig. 11). Each group comprised 8 mice. We divided the mice into two groups of vitamin D-deficiency or vitamin D-repletion by matching the body weight at 12 weeks of age. Further, we divided each group into two groups without or with UV irradiation by matching the serum 25(OH)D level and body weight at 20 weeks of age.

UV irradiation was performed with a 316 nm LED module. The radiation protocol was twice a week with the irradiance intensity and dose determined by the preliminary experiment. The dorsal skins of mice without UV irradiation group were also shaved, irradiated with normal fluorescent light under inhalation anesthesia. Mice were irradiated with UV from 20 to 36 weeks of age. At 36 weeks, they were sacrificed, and samples (femurs, tibias, right kidney, and liver) were obtained, and subjected to the subsequent experiments. During the irradiation period, no apparent complications were observed macroscopically including skin erythema.

#### Serum metabolites

Serum 25(OH)D levels were measured at 12 weeks of age (pre-diet), 20 weeks (pre-UV irradiation), and 28, 36 weeks (8, 16 weeks’ UV irradiation), and 1,25(OH)_2_D levels were measured at 36 weeks. Calcium (Ca) and Inorganic phosphorus (IP) concentrations in serum were measured by standard colorimetric methods using a DryChem (FujiFilm, Tokyo, Japan). They were measured immediately after blood collection. Serum levels of 1–84 parathyroid hormone (PTH) were determined using sandwich ELISA kit (Immutopics, San Clemente, USA). For PTH, sera were stored at − 80 °C until measurement.

#### Real-time RT-PCR analysis

We assayed mRNA expression of enzymes that mediate the metabolic pathway of vitamin D, to assess the effects of UV irradiation on the regulation of the metabolism of vitamin D (25(OH)D and 1,25(OH)_2_D). We determined mRNA levels of C25-hydroxylases (*Cyp2r1, Cyp27a1*) with liver samples and 25-hydroxyvitamin D-1-alpha hydroxylase (*Cyp27b1*) and mRNA levels of 1,25-dihydroxyvitamin D 24-hydroxylase (*Cyp24a1*) with kidney samples by real time RT-PCR. RNA was isolated from liver and kidney of each mouse with the RNeasy Mini Kit (Qiagen, Hilden, Germany) according to the supplier’s protocol. Reverse transcribed cDNA was subjected to real-time RT-PCR using a LightCycler 480 (Roche Diagnostics, Mannheim, Germany), with 480 SYBR Green I Master (Roche Diagnostics, Mannheim, Germany), using 0.5 μM of the sense and antisense specific primers. We applied the conventional amplification program; preincubation step for denaturation of the template cDNA (10 min, 95 °C), followed by 45 cycles of a denaturation step (10 s, 95 °C), an annealing step (10 s, 60 °C), and an extension step (10 s, 72 °C). A negative control without cDNA template was included in every run. To confirm the amplification specificity, the PCR products were subjected to a melting curve analysis on the LightCycler 480 and also a 2% agarose/TAE gel electrophoresis, to measure Tm and amplicon size, respectively. We calculated real-time efficiencies from the given slopes in LightCycler 480 software (Roche Diagnostics, Mannheim, Germany) with dilutions, to allow relative quantification after PCR. The relative levels of mRNAs in a sample were expressed after normalization with those of glyceraldehyde-3-phosphate dehydrogenase (*Gapdh*). The primer pairs for *Gapdh, Cyp27a1, Cyp27b1, and Cyp24a1* were designed according to a previous report^29^. Those for *Cyp2r1* was designed according to another report^30^.

#### Analyses with micro-computed tomography (CT)

Using the distal femur metaphysis, we assessed the influence of UV irradiation on mouse trabecular and cortical microarchitectures. Right femurs were obtained at 36 weeks of age, fixed in 70% ethanol, and subjected to scanning with a high-resolution micro-CT scanner with specific software (SkyScan 1176, Bruker, Kontich, Belgium)^11,31^. Each scan was performed with a rotation step of 0.5° and full rotation of over 180°, with a 0.5 mm aluminum filter for beam-hardening reduction, a source voltage of 50 kV, and current of 500 μA. The exposure time was 0.89 s, and the pixel size was 9 µm. In addition, scans included phantom bones for analysis of bone mineral density (250 mg/cm^3^ and 750 mg/cm^3^) to standardize the grayscale values and maintain consistency between runs. We reconstructed three-dimensional (3D) microstructural images with NRecon software (Bruker, Kontich, Belgium), and calculated morphometric parameters with the SkyScan CT Analyzer (CTAn) software for trabecular and cortical bone in the femur. To determine morphometric parameters of trabecular bone, the volume of interest (VOI) started at 0.17 mm from the growth plate of the femur extending 2 mm toward the diaphysis (2 mm in height) comprising trabecular bone and the marrow cavity. To determine the morphometric parameters of cortical bone, the VOI started at the proximal end of the trabecular VOI extending 2 mm toward the mid shaft (2 mm in height) and comprised the cortical shell only. Bone parameters [bone volume fraction (BV/TV, %), trabecular thickness (Tb.Th, μm), number (Tb.N, 1/mm), spacing (Tb.Sp, mm), bone mineral density (BMD, mg/cm^3^), and cortical thickness (Ct.Th, mm)] were measured according to guidelines for assessing bone microstructure in rodents using micro-CT^32^.

According to a previous study^33^, we assessed body composition using micro-CT scan of total body at 12 and 36 weeks of age for mice while alive. Mice were anesthetized with Isoflurane (2.5% flow rate) and kept under at 2.5% via a nose-cone setup for imaging. Total-body scanning was performed from the first cervical spine to tail root. Each scan was performed with a rotation step of 0.9° and full rotation of over 360°, with a 1 mm aluminum filter for beam-hardening reduction, a source voltage of 50 kV, and current of 500 μA. The exposure time was 0.06 s, and the pixel size was 35 µm. We reconstructed 3D microstructural images with NRecon software, and calculated the fat mass volume and total mass volume with CTAn software for total body. Because of an overlap in density between the lung and fat, the lung area was separated before calculation. The threshold range for total body mass was 30–255 greyscale and that for fat mass was 30–55 greyscale.

#### Grip strength measurement

At 36 weeks of age, grip strength of mouse forefoot was tested using a grip strength meter (Columbus Instruments, OH, USA), and recorded in Newtons (N). Briefly, mice tails were held with the examiner’s fingers, and the mice forearms were allowed to grip the handle. The examiners pulled the mice bodies with their fingers, and pulled them parallel to the floor. Three sets were performed for each mouse, five times as one set, with short breaks between sets. Mean grip strength was calculated. Seven mice in the group of vitamin D-repletion without UV irradiation and 8 mice each in the other groups were evaluated.

#### Mechanical test

The right femur was used to measure the mechanical strength by the 3-point bending test using a mechanical strength analyzer (MZ500D; Maruto, Tokyo, Japan). The femur was placed on a special holding device, and a bending force was applied with the cross head at a speed of 10 mm/min, until fracture occurred. The breaking strength (max load: N) was obtained from the load-deformation curve. Four mice were evaluated in each group.

#### Bone histology

To evaluate new bone formation in undecalcified bone, excised left femurs were analyzed with Villanueva Goldner staining. Specimens at 36 weeks of age were fixed with 70% ethanol for 3 days, dehydrated through a graded ethanol series, and embedded in glycolmethacrylate without decalcification (Aichi Pathologic Laboratory, Aichi, Japan). The embedded tissues were cut into 30-µm coronal sections and examined under a light microscope.

Retrieved left tibias at 36 weeks of age were fixed in 10% formalin and decalcified at 4 °C in 10% EDTA solutions for 2 weeks. To identify osteoclasts, sagittal sections of proximal metaphysis of 5 μm thickness were stained with tartrate-resistant acid phosphatase (TRAP). As a quantitative analysis, the numbers of osteoclasts stained with TRAP in 5 different low-power fields in the proximal metaphysis were counted under a light microscope using ImageJ (National Institutes of Health).

#### Muscle and skin histology

Quadriceps femoris (QF), tibial anterior (TA) and extensor digitorum longus (EDL) were fixed in 10% formalin. After hematoxylin and eosin staining, using ImageJ, diameters of muscle fibers were measured in 30 randomly selected fibers per mouse^13^.

Skin of UV-irradiated areas was retrieved at 36 weeks of age, and fixed in 10% formalin. After hematoxylin and eosin staining, the thickness of epidermis and dermis in ten different fields was measured under a light microscope (magnification × 100)^34^. For immunohistochemical analysis of skin damage, additional tissue sections were stained with rabbit anti-Melan-A monoclonal antibody (ab210546, 1:100 dilution; Abcam, Cambridge, MA, USA) to evaluate melanocytes or keratinocytes with melanin pigmentation, rabbit anti-Cleaved Caspase-3 antibody (ab2302, 1:500 dilution; Abcam, Cambridge, MA, USA) to evaluate apoptosis. These antibodies were confirmed to cross-react with mice antigens with a datasheet provided by the manufacturer. Cells undergoing apoptosis were also identified by the TdT-mediated dUTP nick-end labeling (TUNEL) method with a kit (Apoptosis in situ Detection KIT *Wako*, FUJIFILM Wako Pure Chemical Co., Osaka, Japan). The staining procedure was carried out according to the manufacturer’s recommendation.

### Statistics

The results are presented as mean values ± standard deviation (SD). The Mann–Whitney *U* test and Kruskal–Wallis test were used to compare the results. All statistical analyses were performed using SPSS statistics version 24 (IBM Corp. Armonk, NY, USA). Statistical significance was set at P < 0.05.

## Supplementary information

Supplementary Information 1.

Supplementary Information 2.

Supplementary Information 3.

Supplementary Information 4.

Supplementary Information 5.

## Data Availability

All data generated or analyzed during this study are included in this published article and its Supplementary Information files. Associated protocols and data of this study are available for readers.
