# Supplementary material for: Low energy irradiation of narrow-range UV-LED prevents osteosarcopenia associated with vitamin D deficiency in senescence-accelerated mouse prone 6
Source: Sci Rep. 2020 Jul 17;10:11892. doi: 10.1038/s41598-020-68641-8 (PMC7368004; doi:10.1038/s41598-020-68641-8)
Supplement: Supplementary file 1 — Supplementary Information 1. [file 41598_2020_68641_MOESM1_ESM.docx]

Low energy irradiation of narrow-range UV-LED prevents osteosarcopenia associated with vitamin D deficiency in senescence-accelerated mouse prone 6

Kazuya Makida^1)^, MD, Yoshihiro Nishida*^1)2)^, MD, PhD, Daigo Morita^1)^, MD, PhD, Satoshi Ochiai^1)^, MD, Yoshitoshi Higuchi^1)^, MD, PhD, Taisuke Seki^1)^, MD, PhD, Kunihiro Ikuta^1)^, MD, PhD, Naoki Ishiguro^1)^, MD, PhD

1. Department of Orthopaedic Surgery, Nagoya University Graduate School of Medicine
2. Department of Rehabilitation Medicine, Nagoya University Graduate School of Medicine

**Supplementary Figure Legends**

Suppl. Fig. 1: Levels of *Cyp2r1* mRNAs. Relative expression levels in each group are expressed with reference to that in Vit.D+UV− group as 1.0. Level of Cyp2r1 was normalized with one of *Gapdh* mRNA. The difference was not significant. Vit.D−, vitamin D-deficient diet; Vit.D+, vitamin D-replete diet; UV, ultraviolet irradiation.

Suppl. Fig. 2: Apoptosis markers of UV irradiation on skin tissues. **A–D** Immunohistochemical staining with anti-Cleaved Caspase-3 monoclonal antibody for epidermis and dermis, (original magnification ×10, bars indicate 100 μm). Insets: higher magnification. White arrowheads show Cleaved Caspase-3 positive cells. **E–H** TUNEL staining for epidermis and dermis (original magnification ×100, bars indicate 100 μm). Insets: higher magnification. Positive cells are not seen in all groups. Vit.D−, vitamin D-deficient diet; Vit.D+, vitamin D-replete diet; UV, ultraviolet irradiation; TUNEL, TdT-mediated dUTP nick-end labeling.

Suppl. Fig. 3: Results of histological assays of skin irradiated by short wave-length (305 nm) UV-LED. **A** Hematoxylin and eosin staining for epidermis and dermis, (original magnification ×10, bars indicate 100 μm). The black arrow indicates epidermal-dermal thickness in skin irradiated by short wave-length UV-LED. **B** Immunohistochemical staining with anti-Melan-A monoclonal antibody for epidermis and dermis (original magnification ×100, bars indicate 100 μm). The white arrowheads show a keratinocyte with melanin pigmentation. **C** Immunohistochemical staining with anti-Cleaved Caspase-3 monoclonal antibody for epidermis and dermis, (original magnification ×10, bars indicate 100 μm). Several Cleaved Caspase-3 positive cells could be seen in dermis. **D** TUNEL staining for epidermis and dermis (original magnification ×100, bars indicate 100 μm). Many positive could be seen in epidermis. UV, ultraviolet irradiation; LED, light emitting diode; HE, hematoxylin and eosin staining; Melan, immunohistochemical staining with anti-Melan-A monoclonal antibody; TUNEL, TdT-mediated dUTP nick-end labeling. Insets: higher magnification.

Suppl. Fig. 4: Relative spectral irradiance of a LED module emitting 316 nm wavelength. The wave spectrums and intensity were measured using a UV radiometer.
