# Supplementary figures and images for: Low energy irradiation of narrow-range UV-LED prevents osteosarcopenia associated with vitamin D deficiency in senescence-accelerated mouse prone 6
Source: Sci Rep. 2020 Jul 17;10:11892. doi: 10.1038/s41598-020-68641-8 (PMC7368004; doi:10.1038/s41598-020-68641-8)

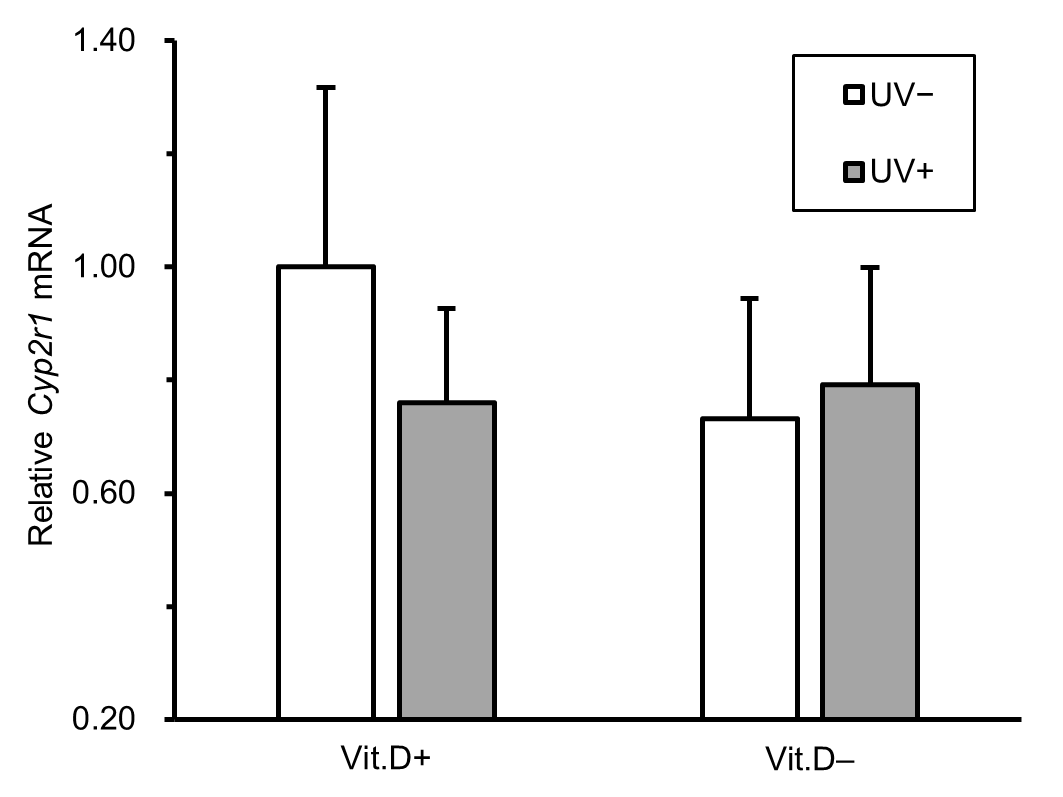

Supplement: Supplementary file 2 — Supplementary Information 2. [file 41598_2020_68641_MOESM2_ESM.tif]

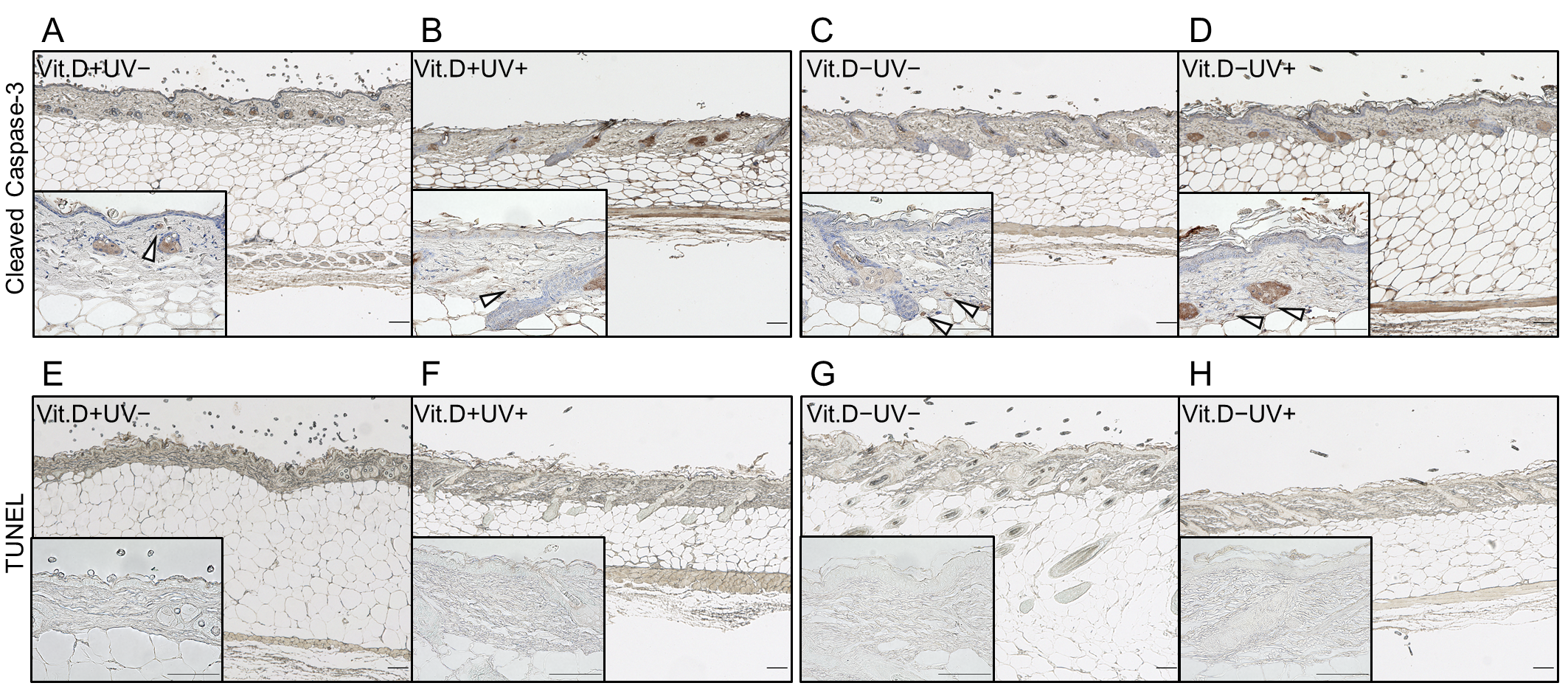

Supplement: Supplementary file 3 — Supplementary Information 3. [file 41598_2020_68641_MOESM3_ESM.tif]

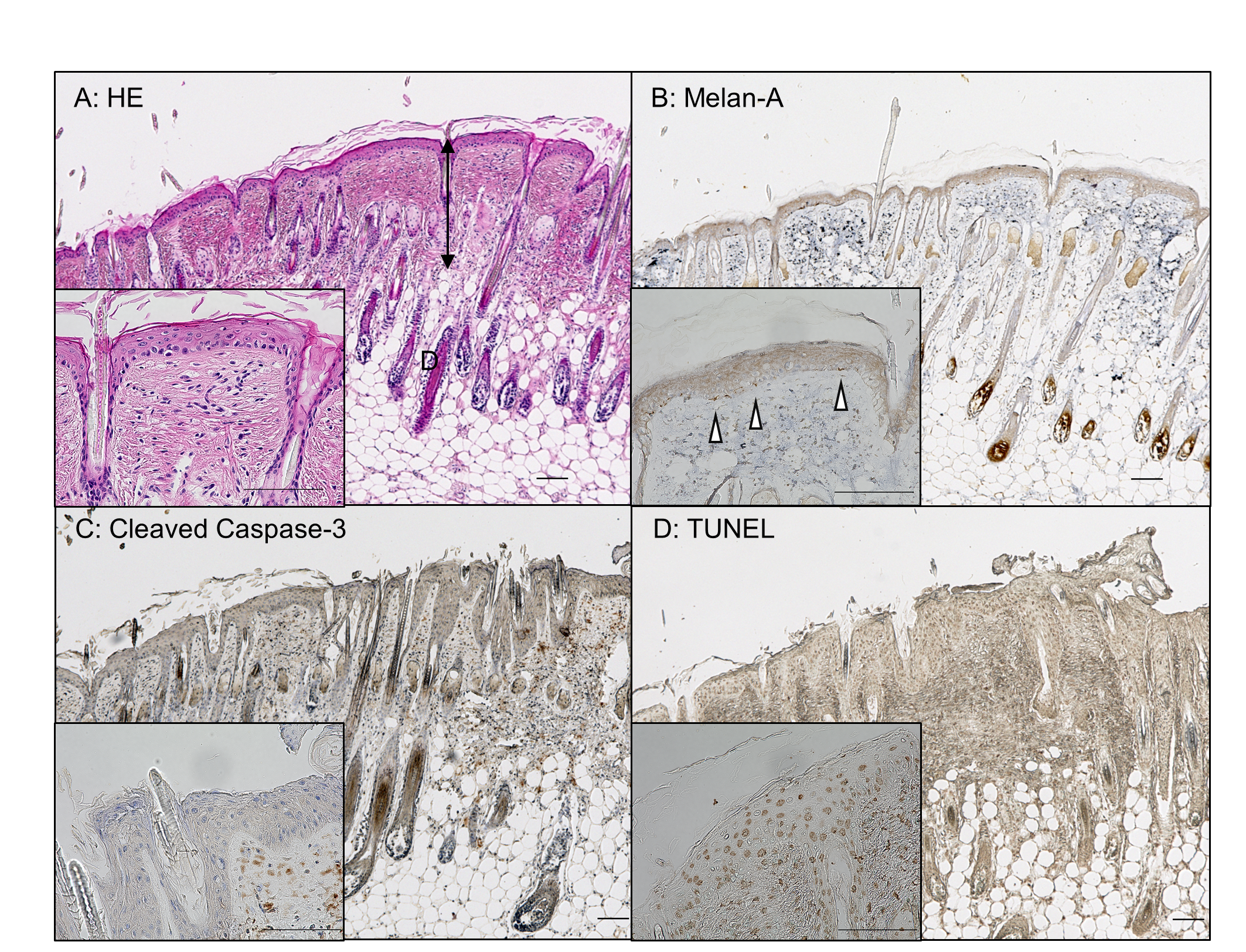

Supplement: Supplementary file 4 — Supplementary Information 4. [file 41598_2020_68641_MOESM4_ESM.tif]

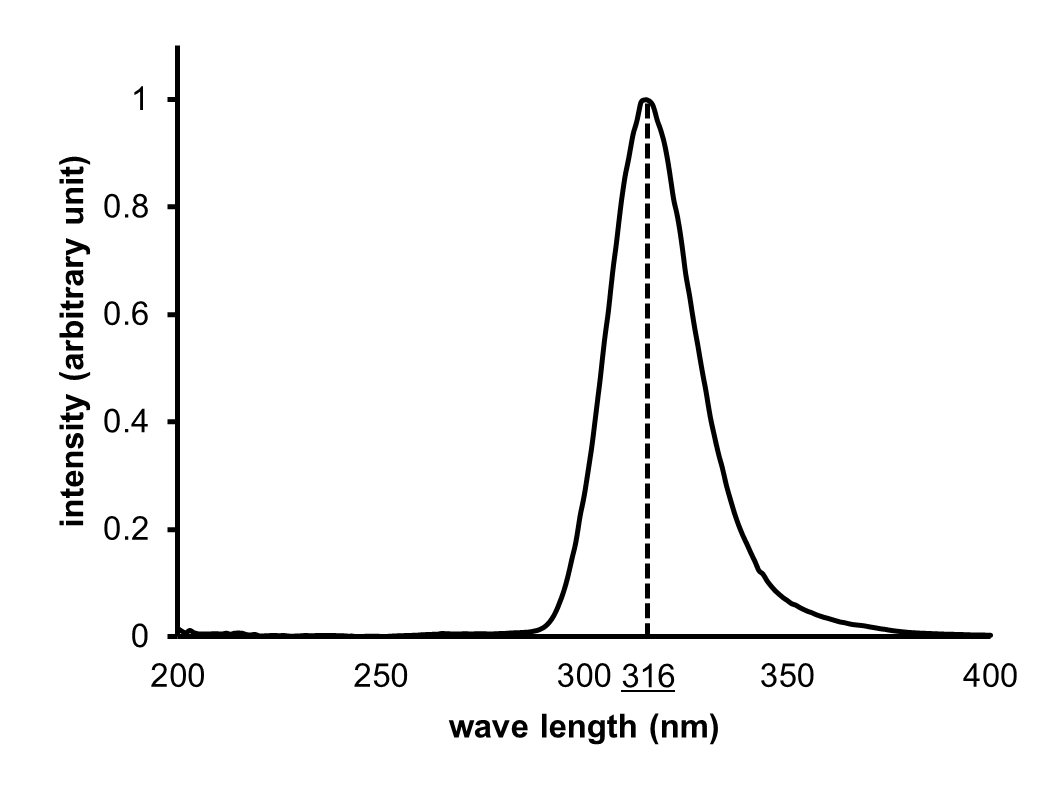

Supplement: Supplementary file 5 — Supplementary Information 5. [file 41598_2020_68641_MOESM5_ESM.tif]
